# Supplementary material for: Identification of photoperiod-induced specific miRNAs in the adrenal glands of Sunite sheep (Ovis aries)
Source: Front Vet Sci. 2022 Jul 22;9:888207. doi: 10.3389/fvets.2022.888207 (PMC9354845; doi:10.3389/fvets.2022.888207)
Supplement: Supplementary file 1 [file Data_Sheet_1.ZIP › Supplementary file/miRDeep2 file.zip!/miRDeep2 file/Know_Structure/LP42A2/oar-mir-23a.pdf]

miRBase precursor : oar-mir-23a
Total read count : 44864
oar-miR-23a read count : 22462
oar-miR-23b read count : 22400
remaining reads : 2

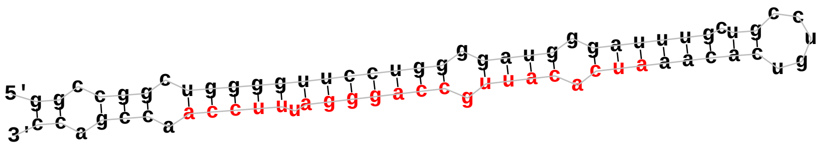

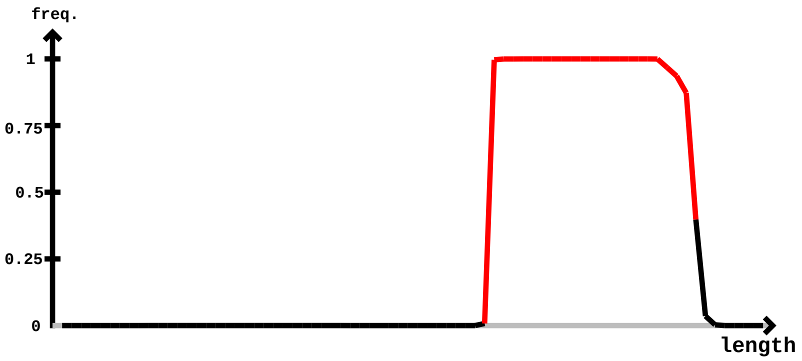


```
                                            oar-miR-23a
                                            oar-miR-23b
5'- ggccggcugggguuccuggggaugggauuugcugccugucacaaaucacauugccagggauuuccaaccgacc  -3'    exp
    ((.(((.((((((((((((.((((.((((((.((.....)))))))).)))).))))))).))))).))).))   reads    mm      sample
    ........gggguuccuggggaugggauu............................................   1        0       seq
    ........gggguuccuggggaugggauuu...........................................   1        0       seq
    ...........................................aaucacauugccagggauu...........   6        0       seq
    ...........................................aaucacauugccagggauuu..........   2        0       seq
    ...........................................aaucacauugccagggauuuc.........   16       0       seq
    ...........................................aaucacauugccagggauuucc........   110      0       seq
    ...........................................aaucacauugccagggauuucca.......   40       0       seq
    ...........................................aaucacauugccagggauuuccaa......   1        0       seq
    ............................................aucacauugccagggau............   9        0       seq
    ............................................aucacauugccagggaC............   1        1       seq
    ............................................aucacauugcUagggauu...........   2        1       seq
    ............................................aucacauugccagggCuu...........   2        1       seq
    ............................................aucacauugccagggauu...........   700      0       seq
    ............................................aucacauugccagggauuu..........   721      0       seq
    ............................................aucacauugccagggauuuc.........   1396     0       seq
    ............................................aucacauugccagggauuucc........   10535    0       seq
    ............................................aucacauugccagggauuucca.......   8050     0       seq
    ............................................aucacauugccagggauuuccaa......   737      0       seq
    ............................................aucacauugccagggauuuccaac.....   58       0       seq
    .............................................ucacauugcUagggauu...........   1        1       seq
    .............................................ucacauugccagggauu...........   2        0       seq
    .............................................ucacauugccagggauuu..........   2        0       seq
    .............................................ucacauugccagggauuuc.........   2        0       seq
    .............................................ucacauugccagggauuucc........   28       0       seq
    .............................................ucacauugccagggauuucca.......   27       0       seq
    .............................................ucacauugccagggauuuccaa......   2        0       seq
    .............................................ucacauugccagggauuuccaac.....   4        0       seq
    ..............................................cacauugccagggauuucc........   1        0       seq
    ...............................................acauugccagggauuucc........   1        0       seq
    ...............................................acauugccagggauuuccU.......   1        1       seq
    ...............................................acauugccagggauuucca.......   3        0       seq
    ...............................................acauugccagggauuuccaa......   1        0       seq
    ................................................cauugccagggauuucc........   1        0       seq
```
